# Supplementary material for: Untrained perceptual loss for image denoising of line-like structures in MR images
Source: PLoS One. 2025 Feb 26;20(2):e0318992. doi: 10.1371/journal.pone.0318992 (PMC11864525; doi:10.1371/journal.pone.0318992)
Supplement: S1 Fig — Illustration of the denoising network ResNet. The residual blocks are repeated five times. The first convolutional layer yields kernel size 9 with 64 output channels. All other convolutional layers yield kernel size 3 and 64 output channels. The last convolutional layer has kernel size 3 and 1 output channel. (PDF) [file pone.0318992.s001.pdf]

Supporting Figure 1

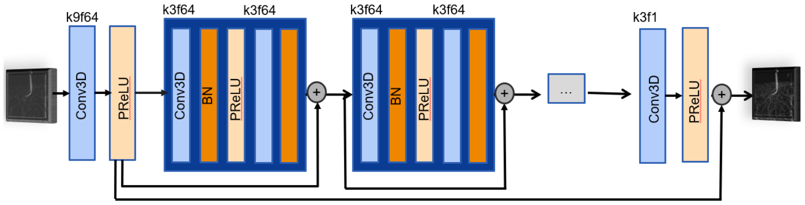

S1 Fig. Illustration of the denoising network ResNet. The residual blocks are repeated five times. The first convolutional layer yields kernel size 9 with 64 output channels. All other convolutional layers yield kernel size 3 and 64 output channels. The last convolutional layer has kernel size 3 and 1 output channel.
