# Supplementary material for: Untrained perceptual loss for image denoising of line-like structures in MR images
Source: PLoS One. 2025 Feb 26;20(2):e0318992. doi: 10.1371/journal.pone.0318992 (PMC11864525; doi:10.1371/journal.pone.0318992)
Supplement: S2 Fig — IIllustration of the denoising Transformer. The Transformer blocks are organized sequentially. Details about the Transformer blocks are explained in the corresponding paper for 2D. (PDF) [file pone.0318992.s002.pdf]

# Supporting Figure 2

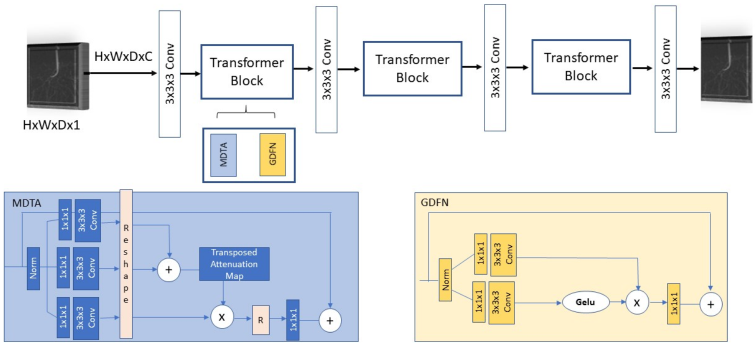


**S2 Fig.** Illustration of the denoising Transformer. The Transformer blocks are organized sequentially. Details about the Transformer blocks are explained in the corresponding paper for 2D.
