# Supplementary figures and images for: Untrained perceptual loss for image denoising of line-like structures in MR images
Source: PLoS One. 2025 Feb 26;20(2):e0318992. doi: 10.1371/journal.pone.0318992 (PMC11864525; doi:10.1371/journal.pone.0318992)

## Supporting Figure 3

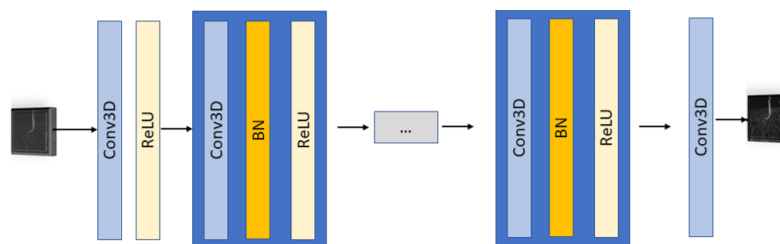

**S3 Fig.** Illustration of the denoising network DnCNN.

Supplement: S3 Fig — Illustration of the denoising network DnCNN. (PDF) [file pone.0318992.s003.pdf]
