# Supplementary material for: Untrained perceptual loss for image denoising of line-like structures in MR images
Source: PLoS One. 2025 Feb 26;20(2):e0318992. doi: 10.1371/journal.pone.0318992 (PMC11864525; doi:10.1371/journal.pone.0318992)
Supplement: S4 Fig — Illustration of cropped image parts on which evaluation metrics were calculated. (PDF) [file pone.0318992.s004.pdf]

Supporting Figure 4

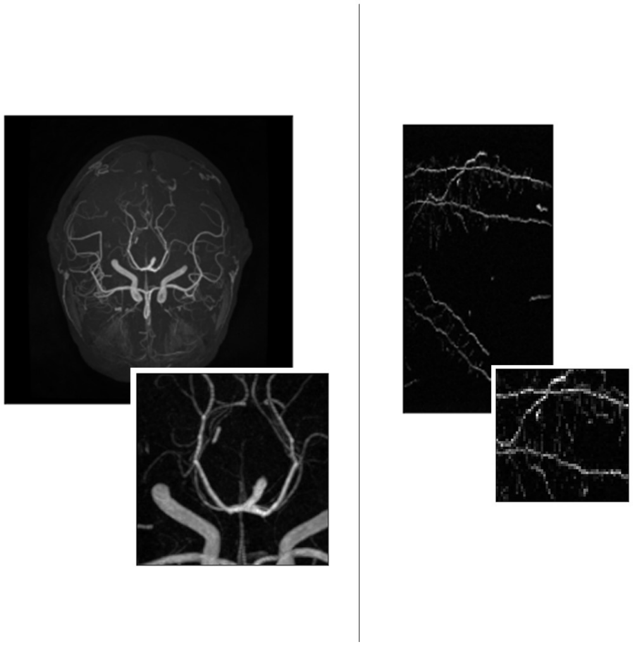

S4 Fig. Illustration of cropped image parts on which evaluation metrics were calculated.
