# Supplementary material for: Untrained perceptual loss for image denoising of line-like structures in MR images
Source: PLoS One. 2025 Feb 26;20(2):e0318992. doi: 10.1371/journal.pone.0318992 (PMC11864525; doi:10.1371/journal.pone.0318992)
Supplement: S1 Table — Evaluation metrics for different random seeds for MRA and root dataset. (PDF) [file pone.0318992.s005.pdf]

Supporting Table 1

Seed	MRA SSIM	MRA PSNR	MRA MSE	MR root SSIM	MR root PSNR	MR root MSE (roots)
1	0.89	32.1	4.2e-3	0.83	37.7	0.033
2	0.90	31.9	4.1e-3	0.84	37.8	0.032
3	0.91	32.0	4.0e-3	0.84	37.9	0.032
4	0.89	32.5	4.3e-3	0.83	37.8	0.032
5	0.90	32.5	4.3e-3	0.84	37.8	0.032

S1 Table. Evaluation metrics for different random seeds for MRA and root dataset.
