# Supplementary material for: Untrained perceptual loss for image denoising of line-like structures in MR images
Source: PLoS One. 2025 Feb 26;20(2):e0318992. doi: 10.1371/journal.pone.0318992 (PMC11864525; doi:10.1371/journal.pone.0318992)
Supplement: S2 Table — Evaluation metrics for MRA images and MR root images for the loss functions included in this study calculated on image parts only. Given are the mean and standard deviation values for the five random seeds used for network training. (PDF) [file pone.0318992.s006.pdf]

Supporting Table 2

Loss	MRA SSIM	MRA PSNR	MRA MSE	MR root SSIM	MR root PSNR	MR root MSE (roots)
L1	0.83 ± 0.01	30.2 ± 0.4	0.0031 ± 6e-4	0.73 ± 0.01	34.2 ± 0.3	0.012 ± 1e-3
SSIM loss	0.82 ± 0.02	31.1 ± 0.8	0.030 ± 0.009	0.74 ± 0.1	34.0 ± 0.7	0.067 ± 0.008
VGG19	0.86 ± 0.02	33.8 ± 0.2	0.0021 ± 0.0007	0.74 ± 0.1	35.19 ± 0.4	0.025 ± 0.006
AlexNet	0.84 ± 0.02	30.9 ± 0.3	0.0033 ± 0.001	0.54 ± 0.3	25.4 ± 0.6	0.051 ± 0.005
SimpleNet	0.87 ± 0.01	33.9 ± 0.3	0.017 ± 7e-4	0.77 ± 0.1	35.5 ± 0.3	0.0023 ± 7e-4

S2 Table. Evaluation metrics for MRA images and MR root images for the loss functions included in this study calculated on image parts only. Given are the mean and standard deviation values for the five random seeds used for network training.
