# Supplementary material for: Untrained perceptual loss for image denoising of line-like structures in MR images
Source: PLoS One. 2025 Feb 26;20(2):e0318992. doi: 10.1371/journal.pone.0318992 (PMC11864525; doi:10.1371/journal.pone.0318992)
Supplement: S3 Table — Mean and std values calculated over the testset for one random seed. Values are similar across random seeds. (PDF) [file pone.0318992.s007.pdf]

Supporting Table 3

Loss	MRA SSIM	MRA PSNR	MRA MSE	MR root SSIM	MR root PSNR	MR root MSE (roots)
L1	0.79 ± 0.06	31.4 ± 1.3	0.0047 ± 0.002	0.79 ± 0.07	37.4 ± 1.5	0.038 ± 0.018
SSIM loss	0.86 ± 0.05	35.6 ± 1.4	0.0051 ± 0.011	0.63 ± 0.03	31.7 ± 1.2	0.058 ± 0.021
VGG19	0.87 ± 0.05	40.2 ± 1.9	0.0075 ± 0.012	0.84 ± 0.07	37.8 ± 1.8	0.031 ± 0.013
AlexNet	0.86 ± 0.06	30.9 ± 1.7	0.0043 ± 0.027	0.75 ± 0.05	28.4 ± 1.3	0.043 ± 0.023
Our SimpleNet	0.90 ± 0.04	41.3 ± 0.9	0.0043 ± 0.0013	0.86 ± 0.05	38.3 ± 0.8	0.021 ± 0.012

S3 Table. Mean and std values calculated over the testset for one random seed. Values are similar across random seeds.
