# Supplementary material for: Untrained perceptual loss for image denoising of line-like structures in MR images
Source: PLoS One. 2025 Feb 26;20(2):e0318992. doi: 10.1371/journal.pone.0318992 (PMC11864525; doi:10.1371/journal.pone.0318992)
Supplement: S4 Table — MSE for different kernel sizes and network depth: MSE calculated only for roots regions (above) and MSE for the whole image also for the MR root dataset (below). (PDF) [file pone.0318992.s008.pdf]

Supporting Table 4

Metric	MSE (roots) - MR root dataset			
	Number of convolutional layers			
Kernel size	3 conv	5 conv	9 conv	13 conv
3	0.031 ± 0.005	0.035 ± 0.004	0.034 ± 0.004	0.032 ± 0.004
5	0.035 ± 0.004	0.038 ± 0.005	0.035 ± 0.005	0.033 ± 0.005
7	0.033 ± 0.006	0.033 ± 0.005	0.033 ± 0.006	0.034 ± 0.006
9	0.033 ± 0.005	0.036 ± 0.006	0.033 ± 0.006	0.034 ± 0.006
	MSE - MR root dataset			
1	7.39e-6 ± 7e-7	7.89e-6 ± 6e-7	9.31e-6 ± 7e-7	9.12e-6 ± 8e-7
3	7.39e-6 ± 6e-7	5.36e-6 ± 7e-7	8.91e-6 ± 6e-7	9.31e-6 ± 8e-7
5	6.41e-6 ± 8e-7	5.75e-6 ± 8e-7	5.67e-6 ± 8e-7	6.12e-6 ± 7e-7
7	5.68e-6 ± 7e-7	5.93e-6 ± 7e-7	6.71e-6 ± 7e-7	6.51e-6 ± 9e-7
9	6.52e-6 ± 7e-7	6.12e-6 ± 6e-7	7.41e-6 ± 8e-7	7.56e-6 ± 9e-7

S4 Table. MSE for different kernel sizes and network depth: MSE calculated only for roots regions (above) and MSE for the whole image also for the MR root dataset (below).
