# Supplementary material for: Untrained perceptual loss for image denoising of line-like structures in MR images
Source: PLoS One. 2025 Feb 26;20(2):e0318992. doi: 10.1371/journal.pone.0318992 (PMC11864525; doi:10.1371/journal.pone.0318992)
Supplement: S5 Table — PSNR/MSE for different kernel sizes and network depth for MRA (above) and MSE for the MRA dataset (below). (PDF) [file pone.0318992.s009.pdf]

## Supporting Table 5

| Metric | PSNR - MRA dataset | | | |
|---|---|---|---|---|
| | Number of convolutional layers | | | |
| Kernel size | 3 conv | 5 conv | 9 conv | 13 conv |
| 3 | 41.3 ± 0.2 | 41.2 ± 0.2 | 40.0 ± 0.3 | 40.7 ± 0.4 |
| 5 | 41.1 ± 0.3 | 41.6 ± 0.3 | 39.3 ± 0.4 | 40.0 ± 0.3 |
| 7 | 40.2 ± 0.3 | 39.4 ± 0.4 | 38.4 ± 0.3 | 39.5 ± 0.5 |
| 9 | 41.1 ± 0.3 | 39.2 ± 0.3 | 40.3 ± 0.4 | 39.5 ± 0.5 |
| | MSE - MRA dataset | | | |
| 3 | 0.0043 ± 0.0004 | 0.0047 ± 0.0004 | 0.0072 ± 0.0005 | 0.0071 ± 0.0006 |
| 5 | 0.0051 ± 0.0006 | 0.0083 ± 0.0004 | 0.0041 ± 0.0004 | 0.0041 ± 0.0003 |
| 7 | 0.0043 ± 0.0005 | 0.0088 ± 0.0006 | 0.0052 ± 0.0005 | 0.0051 ± 0.0004 |
| 9 | 0.0042 ± 0.0004 | 0.0071 ± 0.0005 | 0.0046 ± 0.0006 | 0.0045 ± 0.0004 |

**S5 Table.** PSNR/MSE for different kernel sizes and network depth for MRA (above) and MSE for the MRA dataset (below).
