# Supplementary material for: Untrained perceptual loss for image denoising of line-like structures in MR images
Source: PLoS One. 2025 Feb 26;20(2):e0318992. doi: 10.1371/journal.pone.0318992 (PMC11864525; doi:10.1371/journal.pone.0318992)
Supplement: S6 Table — PSNR values for both datasets, network structures, and noise levels. (PDF) [file pone.0318992.s010.pdf]

## Supporting Table 6

| | PSNR - MR root dataset | | | |
|---|---|---|---|---|
| Network/Loss | 1 % noise | 5 % noise | 10 % noise | 20 % noise |
| DnCNN/L1 | 36.3 ± 0.3 | 35.0 ± 0.3 | 34.4 ± 0.5 | 34.0 ± 0.4 |
| DnCNN/uPL | 37.2 ± 0.2 | 35.9 ± 0.3 | 35.3 ± 0.2 | 34.8 ± 0.3 |
| ResNet/L1 | 36.2 ± 0.1 | 35.1 ± 0.2 | 35.0 ± 0.2 | 34.7 ± 0.2 |
| ResNet/uPL | 36.4 ± 0.3 | 36.0 ± 0.3 | 35.7 ± 0.3 | 35.0 ± 0.3 |
| Transformer/L1 | 34.3 ± 0.2 | 32.3 ± 0.3 | 30.9 ± 0.2 | 29.5 ± 0.3 |
| Transformer/uPL | 35.7 ± 0.2 | 34.3 ± 0.3 | 33.2 ± 0.4 | 30.0 ± 0.3 |
| | PSNR - MRA dataset | | | |
| DnCNN/L1 | 0.86 ± 0.01 | 0.81 ± 0.01 | 31.4 ± 0.41 | 0.81 ± 0.01 |
| DnCNN/uPL | 0.92 ± 0.01 | 0.87 ± 0.01 | 0.87 ± 0.01 | 0.84 ± 0.02 |
| ResNet/L1 | 47.1 ± 1.6 | 40.1 ± 0.6 | 39.9 ± 0.6 | 41.2 ± 0.6 |
| ResNet/uPL | 44.2 ± 0.6 | 43.1 ± 1.2 | 42.6 ± 0.8 | 40.0 ± 0.5 |
| Transformer/L1 | 0.97 ± 0.03 | 0.91 ± 0.02 | 0.85 ± 0.02 | 0.68 ± 0.03 |
| Transformer/uPL | 0.98 ± 0.02 | 0.93 ± 0.01 | 0.83 ± 0.01 | 0.78 ± 0.02 |

**S6 Table.** PSNR values for both datasets, network structures, and noise levels.
