# Supplementary material for: Untrained perceptual loss for image denoising of line-like structures in MR images
Source: PLoS One. 2025 Feb 26;20(2):e0318992. doi: 10.1371/journal.pone.0318992 (PMC11864525; doi:10.1371/journal.pone.0318992)
Supplement: S7 Table — MSE values only calculated on the roots part and MSE values for both datasets for all network architectures, and noise levels included in this study. (PDF) [file pone.0318992.s011.pdf]

Supporting Table 7

Network/Loss	1 % noise	5 % noise	10 % noise	20 % noise
	MSE - MR root dataset			
DnCNN/L1	5.78e-6 ± 1e-7	6.21e-7 ± 5e-7	7.79e-6 ± 8e-6	7.80e-6 ± 3e-7
DnCNN/uPL	5.35e-6 ± 6e-7	5.99e-6 ± 5e-7	7.39e-6 ± 7e-7	7.73e-6 ± 5e-7
ResNet/L1	5.92e-6 ± 7e-7	6.51e-7 ± 4e-7	7.68e-6 ± 4e-6	8.01e-6 ± 5e-7
ResNet/uPL	5.45e-6 ± 4e-7	6.14e-7 ± 2e-7	6.98e-7 ± 8e-7	7.63e-6 ± 3e-6
Transformer/L1	5.51e-6 ± 6e-7	5.9e-6 ± 2e-7	7.5e-6 ± 2e-7	7.6e-6 ± 3e-7
Transformer/uPL	5.51e-6 ± 5e-7	5.64e-6 ± 3e-7	6.9e-6 ± 8e-7	7.3e-6 ± 3e-7
	MSE (roots) - MR root dataset			
DnCNN/L1	0.012 ± 5e-3	0.034 ± 8e-3	0.031 ± 4e-3	0.036 ± 3e-3
DnCNN/uPL	0.010 ± 6e-3	0.032 ± 2e-3	0.032 ± 6e-3	0.032 ± 7e-3
ResNet/L1	0.013 ± 7e-3	0.033± 8e-3	0.034 ± 6e-3	0.034 ± 8e-3
ResNet/uPL	0.0098 ± 6e-3	0.031 ± 3e-3	0.032 ± 5e-3	0.033 ± 6e-3
Transformer/L1	0.012 ± 2e-3	0.035 ± 9e-3	0.036 ± 6e-3	0.036 ± 8e-3
Transformer/uPL	0.010 ± 4e-3	0.032 ± 5e-3	0.033 ± 6e-3	0.034 ± 5e-3
	MSE - MRA dataset			
DnCNN/L1	4.1e-3 ± 6e-4	4.7e-3 ± 4e-4	0.034 ± 0.004	5.9e-3 ± 5e-4
DnCNN/uPL	3.8e-3 ± 5e-4	4.1e-3 ± 5e-4	4.9e-3 ± 4e-4	5.3e-3 ± 5e-4
ResNet/L1	3.2e-3 ± 5e-4	4.8e-3 ± 3e-4	5.9e-3 ± 7e-4	5.1e-3 ± 6e-4
ResNet/uPL	3.9e-3 ± 4e-4	4.5e-3 ± 2e-4	4.9e-3 ± 6e-4	5.7e-3 ± 5e-4
Transformer/L1	3.8e-3 ± 5e-4	4.3e-3 ± 8e-4	5.7e-3 ± 5e-4	6.2e-3 ± 3e-4
Transformer/uPL	3.1e-3 ± 4e-4	4.1e-3 ± 5e-4	6.1e-3 ± 7e-4	6.2e-3 ± 8e-4

S7 Table. MSE values only calculated on the roots part and MSE values for both datasets for all network architectures, and noise levels included in this study.
