# Supplementary material for: Untrained perceptual loss for image denoising of line-like structures in MR images
Source: PLoS One. 2025 Feb 26;20(2):e0318992. doi: 10.1371/journal.pone.0318992 (PMC11864525; doi:10.1371/journal.pone.0318992)
Supplement: S8 Table — SSIM values for both datasets calculated on the center of the image. (PDF) [file pone.0318992.s012.pdf]

Supporting Table 8

	SSIM - Image Part MR root			
Network/Loss	1 % noise	5 % noise	10 % noise	20 % noise
DnCNN/L1	0.80 ± 0.01	0.77 ± 0.01	0.73 ± 0.01	0.74 ± 0.01
DnCNN/uPL	0.85 ± 0.01	0.84 ± 0.01	0.77 ± 0.01	0.76 ± 0.01
ResNet/L1	0.81 ± 0.02	0.77 ± 0.01	0.75 ± 0.01	0.74 ± 0.01
ResNet/uPL	0.85 ± 0.01	0.83 ± 0.01	0.78 ± 0.01	0.78 ± 0.01
Transformer/L1	0.77 ± 0.02	0.75 ± 0.01	0.67 ± 0.02	0.46 ± 0.03
Transformer/uPL	0.81 ± 0.01	0.79 ± 0.01	0.77 ± 0.01	0.66 ± 0.02
	SSIM - Image Part MRA			
DnCNN/L1	0.95 ± 0.01	0.87 ± 0.01	0.83 ± 0.01	0.83 ± 0.02
DnCNN/uPL	0.97 ± 0.01	0.92 ± 0.01	0.87 ± 0.01	0.86 ± 0.01
ResNet/L1	0.98 ± 0.01	0.92 ± 0.01	0.87 ± 0.01	0.84 ± 0.01
ResNet/uPL	0.98 ± 0.01	0.95 ± 0.01	0.89 ± 0.01	0.87 ± 0.01
Transformer/L1	0.90 ± 0.01	0.86 ± 0.01	0.84 ± 0.01	0.78 ± 0.01
Transformer/uPL	0.95 ± 0.01	0.89 ± 0.01	0.83 ± 0.01	0.83 ± 0.01

S8 Table. SSIM values for both datasets calculated on the center of the image.
