# Supplementary material for: Untrained perceptual loss for image denoising of line-like structures in MR images
Source: PLoS One. 2025 Feb 26;20(2):e0318992. doi: 10.1371/journal.pone.0318992 (PMC11864525; doi:10.1371/journal.pone.0318992)
Supplement: S9 Table — PSNR values for both datasets calculated on the center of the image. (PDF) [file pone.0318992.s013.pdf]

Supporting Table 9

Network/Loss	1 % noise	5 % noise	10 % noise	20 % noise
	PSNR - Image Part MR root			
DnCNN/L1	35.6 ± 0.2	35.5 ± 0.4	34.2 ± 0.3	34.0 ± 0.2
DnCNN/uPL	35.7 ± 0.3	34.8 ± 0.2	35.5 ± 0.4	34.3 ± 0.3
ResNet/L1	35.7 ± 0.2	34.3 ± 0.2	35.3 ± 0.2	33.3 ± 0.3
ResNet/uPL	36.2 ± 0.2	35.9 ± 0.3	35.5 ± 0.3	34.3 ± 0.3
Transformer/L1	35.4 ± 0.2	31.3 ± 0.3	29.8 ± 0.4	27.7 ± 0.4
Transformer/uPL	35.8 ± 0.2	34.3 ± 0.4	31.3 ± 0.3	29.8 ± 0.3
	PSNR - Image Part MRA			
DnCNN/L1	37.0 ± 0.2	32.8 ± 0.3	30.2 ± 0.4	28.0 ± 0.4
DnCNN/uPL	37.4 ± 0.3	33.4 ± 0.2	33.9 ± 0.3	30.1 ± 0.2
ResNet/L1	37.3 ± 0.2	37.0 ± 0.2	34.2 ± 0.3	28.2 ± 0.4
ResNet/uPL	39.3 ± 0.2	38.6± 0.3	37.8 ± 0.3	32.1 ± 0.3
Transformer/L1	39.1 ± 0.4	38.0 ± 0.3	32.2 ± 0.3	26.2 ± 0.3
Transformer/uPL	39.0 ± 0.3	37.5 ± 0.2	34.3 ± 0.2	32.2 ± 0.4

S9 Table. PSNR values for both datasets calculated on the center of the image.
