# Supplementary material for: Untrained perceptual loss for image denoising of line-like structures in MR images
Source: PLoS One. 2025 Feb 26;20(2):e0318992. doi: 10.1371/journal.pone.0318992 (PMC11864525; doi:10.1371/journal.pone.0318992)
Supplement: S10 Table — MSE values for both datasets calculated on the center of the image. (PDF) [file pone.0318992.s014.pdf]

Supporting Table 10

	MSE (roots) - Image Part MR root			
Network/Loss	1 % noise	5 % noise	10 % noise	20 % noise
DnCNN/L1	0.008 ± 7e-4	0.012 ± 1e-3	0.012 ± 1e-3	0.019 ± 1e-3
DnCNN/uPL	0.006 ± 8e-4	0.009± 9e-4	0.015 ± 7e-4	0.017 ± 1e-4
ResNet/L1	0.007 ± 6e-4	0.009 ± 8e-4	0.010 ± 7e-4	0.011 ± 7e-4
ResNet/uPL	0.006 ± 8e-4	0.007 ± 1e-3	0.008 ± 8e-4	0.008 ± 8e-4
Transformer/L1	0.010 ± 9e-4	0.018 ± 2e-3	0.019 ± 1e-3	0.021 ± 2e-3
Transformer/uPL	0.011 ± 8e-4	0.012 ± 1e-3	0.014 ± 2e-3	0.019 ± 2e-3
	MSE - Image Part MRA			
DnCNN/L1	0.0017 ± 6e-4	0.0025 ± 7e-4	0.0031 ± 6e-4	0.0033 ± 8e-4
DnCNN/uPL	0.0016 ± 5e-4	0.0019 ± 8e-4	0.0023 ± 7e-4	0.0025 ± 9e-4
ResNet/L1	0.0013 ± 7e-4	0.0021 ± 6e-4	0.0024 ± 7e-4	0.0025 ± 7e-4
ResNet/uPL	0.0011 ± 5e-4	0.0017 ± 8e-4	0.0020 ± 5e-4	0.0021 ± 8e-4
Transformer/L1	0.0015 ± 4e-4	0.0019 ± 5e-4	0.0039 ± 4e-4	0.0043 ± 6e-4
Transformer/uPL	0.0016 ± 6e-4	0.0017 ± 6e-4	0.0021 ± 9e-4	0.0022 ± 9e-4

S10 Table. MSE values for both datasets calculated on the center of the image.
