# Supplementary material for: Untrained perceptual loss for image denoising of line-like structures in MR images
Source: PLoS One. 2025 Feb 26;20(2):e0318992. doi: 10.1371/journal.pone.0318992 (PMC11864525; doi:10.1371/journal.pone.0318992)
Supplement: Attachment — Submitted filename: AnswerToReviewers_PLOS.docx [file pone.0318992.s015.docx]

We thank the reviewers for their valuable feedback which helped to increase the quality of the manuscript. We adapted the manuscript accordingly. Our detailed answers are listed below.

_______________________________________________________________________________________

Reviewer #1: 

1. The evaluation metrics used in this paper are predominantly influenced by large structures in the image. However, the regions that need to be restored through denoising are the fine structures. Therefore, the evaluation results for the fine structure areas (for example, the left half of the image in Fig. 2) should be presented alongside the evaluation results for the entire image.

*We thank the reviewer for this important point. We now also calculated the image quality metrics for the cropped region of the images. All these regions also contained vessels/roots (i.e. the important parts of the images), we calculated the image quality metrics also on these regions. To avoid confusion, we added the results to the supplemental information. We added the following sentence to the manuscript (p.6, l.109):*

*‚As both datasets contain fine structures, the evaluation metrics were additionally calculated on the parts of the image containing important information. A cube of size 52 x 52 x 52 was cropped from the root data. As the roots grow from up to down, the cube was cropped from the upper middle part of the image. A cube of size 68 x 68 x 68 was cropped from the middle of the MRA images as these images contain important information mainly in the image center. An example illustration is displayed in Supplemental Figure S2 . These results are reported in the Supplemental Information.‘*

*We observed different, but consistent results when calculating evaluation metrics on the whole image and on image parts only (i.e. for the root dataset, 10% noise, and DnCNN network: L1 Loss SSIM: 0.73, uPL: SSIM 0.77 when calculated on image parts; fort he MRA dataset, 10% noise and DnCNN network: L1: SSIM 0.83, uPL: 0.87)*

2. Line 254: uPL takes neighborhood information into account when calculating loss. What is the basis for this claim?

*As the uPL compares feature maps that are acquired by applying convolutional kernels with a kernel size > 1, these feature maps also contain information about the voxel neighborhood. We added the following sentence to the manuscript for clarification (p.11, l300): ‚This is probably because the uPL is taking neighborhood information into account by using convolutional kernels with sizes larger than 1 when calculating the loss.‘*

3. It is thought that the effectiveness of perceptual loss arises because the trained network extracts meaningful feature maps. There have been papers that trained a network specifically to extract meaningful features for the primary task, and these papers indeed improved the performance of the primary task. Therefore, a discussion is needed on why an untrained network is also effective.

*Thank you for this question. We added a part to the discussion responding this question. It reads (p11, l.313): ‚* *The success of perceptual loss was thought to be due to the features networks learned during training. However, as previous studies demonstrated for 2D images, pre-trained and untrained networks used in the loss function lead to comparable results (Liu et al., Generic perceptual loss for modeling structured output dependencies, Proceedings of the IEEE/CVF Conference on Computer Vision and Pattern Recognition). This might be because randomly initialized weights can also represent the statistical properties of the training data. The pre-trained networks are usually trained on classification tasks. However, other image characteristics could be more important for image denoising of an entire image. E.g. the display of sharp edges can also be captured by convolutions and untrained feature maps. Additionally, the perceptual distance between two images is equivalent to the maximum mean discrepancy (MMD) distance between local distributions of small patches in the two images (Amir and Weiss, Understanding and Simplifying Perceptual Distances, CVPR 2021). As also demonstrated in this study, different network structures in the loss function have a high impact on the denoising results. These findings suggest that the design of the network architecture used in the loss function can be chosen such that it captures the most important image information. ‘*

Reviewer #2: The manuscript describes a new deep learning approach to the denoising of magnetic resonance (MR) images, especially those depicting fine fiber structures. The core idea is to use an “untrained perceptual loss” (uPL) to drive the training of the deep neural networks (DNN) intended for denoising. This approach is not new, but here applied and benchmarked systematically for 3D MR image stacks for the first time. The results show that the uPL loss is actually superior to other commonly used loss functions in this domain, at least for the chosen datasets and denoising network architectures.

===============================================
Overall assessment
===============================================

The scientific idea and results are definitely worth publishing because they provide some new unique insights. However, the writing needs some improvement (more on this in the next paragraph), and the presented experiments seem to be carried out only once for every task condition. Thus, the results which we find in the tables are derived from a single training run of a single network. We all know that DNN training is a stochastic process where the outcome can vary from training run to training run. Therefore, I strongly recommend to carry out multiple training runs per task condition and to present the mean outcome in the tables (incl. standard deviation).

*We thank the reviewer for pointing this out. We now ran the networks for each condition five times (with five different random seeds). We report the mean and std values across random seeds in the Tables. We added the following sentence to the Materials and Methods section: ‚All experiments were performed with five different random seeds*.‘

Even with the current data, one should report for every value in the result tables the standard deviations related to the variability in the test set and the number of test samples. In this way it would be possible to estimate if any of the observed differences are significant in a statistical sense. Even better would be proper statistical testing by the authors to convince the reader that the reported differences in the various performance measures are meaningful at all. For now, in my current assessment of the paper, I *assume* that this is the case for most reported differences in SSIM, PSNR, and MSE values.

*We thank the reviewer for pointing this out; We added the standard deviation across random seeds to the results. The results for one random seed including the standard deviation in the testset is separately displayed in the supplemental material. However, we hope and believe with the application of five random seeds and the additional evaluation on image parts only (requested by Reviewer 1), our results are convincing.*

The abstract and introduction section are written very well. For the first paragraph of the introduction, the following reference may be a good addition: ZH Shah, M Müller, B Hammer, T Huser, and W Schenck. Impact of different loss functions on denoising of microscopic images. In 2022 International Joint Conference on Neural Networks (IJCNN), 2022.
(in this paper, various loss functions for denoising are compared with each other, among them a version of the perceptual loss; to include this reference is just a suggestion because it seems to be a good fit, but it is not a must)

*We thank the reviewer for mentioning this paper which is indeed a very good fit. We added it to the introduction section (p2, l.30): ‚The Perceptual Loss is also often used in combination with L1- or L2-loss where it also leads to performance improvements ‘*

The section on “Materials and Methods” lacks some clarity, however. Starting at the subsection “Conventional loss functions”, the reader gets easily confused how the different uPL variations are related to each other. First, VGG19, AlexNet and a simple CNN are mentioned. It is not explicitly written that only the simple CNN serves as starting point for the variations of weight initialization, depth, and number of pooling layers. It is also not clear from the writing how the latter three variations interact with each other.

*We adapted ‚Materials and Methods‘ section accordingly. We changed the order of the subsections: We now first introduce the untrained perceptual loss and then introduce a simple ‚proof of concept‘ study where we compare the performance of a simple uPL network with the performance of other networks. We then elaborate on the impact of different uPL network characteristics on the results. (p.4ff)*

Regarding the training details, the question is why 30.000 iterations were chosen. Is this the optimum number for the performance measures on the validation sets? No over- or underfitting in any of the experimental variations?
Also the learning rate of 0.001 needs justification.

*We experimented with different learning rates and numbers of iterations. The chosen settings led to the overall optimal performance in the validation sets. As performance was similar in the training set, we assume no over- or underfitting occured for this numbers. We added the following part to the manuscript (p6 l.181):*

*‚The training parameters were chosen as they lead to the overall best performance in the validation set.‘*

The writing in the “Results” section should also be improved because it is partly confusing. First, I strongly recommend to check and improve grammar and sentence structure, second, to state in the beginning of each subsection what is the goal of the described experiment and what is the experimental configuration (because it does not always fully align with the corresponding part in the “Methods” section).

*We thank the reviewer for this important point. We changed the wording and structure of this section accordingly. We made some small changes in each part and added a sentence describing the aim of each section (p9ff).*

Also reg. the “Results” section, I recommend to add more visualizations of the denoising results. The existing figures and possible additional figures could be formatted in a nicer and more compact way.

*We changed the Figures and added some more visual results.*

The “Discussion” section is appropriate.


===============================================
Small remarks
===============================================

• writing style not consistent reg. “2D” vs. “2d” and “3D” vs. “3d”

*Thank you for pointing this out. We changed all namings now to 2D and 3D.*

• writing style not consistent reg. “transformer” vs. “Transformer”

*Done*
• p. 4, beginning: No figure for DuCNN in suppl. Materials

*Done*
• p. 4, 3rd paragraph: More in-depth explanation about adaption of transformer architecture to 3D setting would be nice

*We added a more detailed explanation. Additionally, we changed the Supplemental Figure and added more details. The section reads now (p4, l.94ff)*:

*The Transformer network is specially designed such that it can be used for large images while modeling global connectivity. In this network, multi-head ’transposed’ attention (MDTA) blocks are introduced applying attention across feature dimensions rather than across spatial dimensions. Before feeding the data to the MDTA blocks, the images are resized to the size (image height · image width) × number of channels. After the MDTA blocks follow feed-forward blocks consisting of two convolutional layers and a gating layer. We adjust the transposition in the attention blocks to the 3D case. In the 3D Transformer network, all 2D convolutions are replaced by 3D convolutions. Before the data is fed to the MDTA blocks, the data is resized to the size (image height· image width · image depth) × number of channels. As for our fine structures, UNet-like architectures lead to performance drops, the Transformer blocks were in our study combined sequentially. A graphical overview of all network structures is displayed in the supplemental material (S1 Fig, S2 Fig, S3 Fig).*

• p. 6, line 190-192: If uniform init. is worse than Xavier normal, why use default init. in “all other experiments”?

*We apologize, this was a mistake. We trained all networks with xavier normal initialisation. We changed the sentence in the manuscript accordingly.*

• line 195: “slighlty” --> “slightly”

*Done.*

• caption of table 3: “… MRA (above) for…” --> “… MRA (above) and for…”

*Done*

• line 201: “…show the number…” --> “…show that the number…”

*Done*

• line 204-208: Description of the results for MRA does not reflect the similarly good results for 7 conv. Layers

*Done*

• caption of Fig. 3 reg. “difference images”: Difference to what?

TODO
• line 226/227: I don’t understand this sentence since the results in table 4 show consistently better results for uPL at the highest noise level with the roots dataset.

*True. We changed the sentence accordingly to:*  *For the MRA data, the superiority in performance was not visible for the lowest and highest noise level and the ResNet, and 10 % added noise and the Transformer network.*

• Caption of table 4: There are no other eval. metrics listed in the suppl. materials section reg. table 4 (btw, what is “Section 7”?)

*Apologize for this mistake. We added the tables to the Supplemental information.*

• Figures S2 and S3: Not very helpful for better understanding (esp. S2)

*We changed the corresponding figures accordingly.*

• Figure S4: Subfigure (g) obviously missing

*Done*
• Caption of Table S2: Very confusing

*We changed the caption to: MSE for different kernel sizes and network depth: MSE calculated only for roots regions (above) and MSE for the whole image also for the MR root dataset (below)*

• Caption of Table S3: Replace “SSIM” with “PSNR/MSE”

*Done.*
