# Supplementary material for: Untrained perceptual loss for image denoising of line-like structures in MR images
Source: PLoS One. 2025 Feb 26;20(2):e0318992. doi: 10.1371/journal.pone.0318992 (PMC11864525; doi:10.1371/journal.pone.0318992)
Supplement: Attachment — Submitted filename: Reviewer Attachment.docx [file pone.0318992.s016.docx]

**Manuscript Number:** PONE-D-24-26390R1
**Manuscript Title:** Untrained Perceptual Loss for image denoising of line-like structures in MR images

The topic is relevant and may be of interest to a broad range of the journal's readers. However, this reviewer has some major concerns about the paper.

1) The abstract does not highlight the specifics of the research or findings but contains too much background information. It is good to provide some specifics (e.g., sample size, dataset size, numbers from results, etc.).

2) The discussion of the results does not highlight the strengths and weaknesses of the proposed approach.

3) I suggest adding a clear research objective and research questions in the introduction section and specify what the main research problem or hypothesis is addressed.

4) Please add more recent references. Certainly, there has been more recent (within the last two years) research on this topic published in information science and/or computer science outlets. An academic search on the topic (using keywords from the manuscript's title) shows that there is recent work in this area. Therefore, authors must update their literature review.

5) Starting from the previous works, I suggest introducing a table to summarize the most recent works and to highlight the novelty of the proposed work.

6) The manuscript only provides the structure of network, it is necessary to explain why these networks can work effectively and to show the concrete parameters.

7) Although a novel approach might be allowed, it is required to provide technical details of the proposed methods as much as possible and in-depth explanations of method selection process.

8) While acquiring the image sometimes the quality may be poor due to blurring which may be caused due to motion artefact. How this problem may be tackled in the proposed scheme?

9) The authors should need to site all equations and Figures in text.

10) It is suggested to conduct statistical tests. For example, see Section 4.5 in paper [a].

[a] Biswas, S., Mostafiz, R., Uddin, M.S. and Paul, B.K., 2024. XAI-FusionNet: Diabetic foot ulcer detection based on multi-scale feature fusion with explainable artificial intelligence. *Heliyon*, *10*(10), p.e31228.

11) I think, the authors should introduce a comparison table to compare the proposed work with other existing works.

12) The reference list needs tidying up, as there are references missing items or formatting issues. Please be consistent with the formatting and use some standard formatting style.
