# Supplementary material for: Untrained perceptual loss for image denoising of line-like structures in MR images
Source: PLoS One. 2025 Feb 26;20(2):e0318992. doi: 10.1371/journal.pone.0318992 (PMC11864525; doi:10.1371/journal.pone.0318992)
Supplement: Attachment — Submitted filename: plos_answer_HS.docx [file pone.0318992.s017.docx]

***We thank the reviewers for their valuable time and feedback which helped to improve the manuscript. Detailed answers are given below.***

***_________________________________________________________***

Reviewer 3:

I think the figure and table captions need revision. They are not very clear and are missing information.

The discussion could be revised. It is a bit anemic. I would include a brief discussion of the limitations of your approach. I would also consider mentioning the MRA results, since you mention the root results.

*We thank the reviewer for this comment. We added the following part to the discussion: ‚Results are considerably better for the MRA dataset. With SSIM values between 0.99 and 0.93 for the lower noise levels, the proposed networks might be used for image denoising in a clinical setting. However, to apply the proposed approach in a clinical setting, our denoising framework needs to be verified on MRA data including healthy and unhealthy subjects (i.e. patients with e.g. an aneurysm). In a clinical scenario, it needs to be verified that denoising does not suppress important clinical information. This will be done in future studies based on this work.’*

*Moreover, we included a paragraph about the limitations of our study. ‚Our study has several limitations. First, all networks were trained and applied on data acquired at the same scanner. In future work, we will train and test our approach on data from different scanners yielding different image characteristics. Second, the MRA dataset used in this study yields exclusively images from healthy subjects. In future work, we will apply the networks also to subjects with vessel abnormalities.*

*MR images of human subjects also suffer from motion artifacts. The proposed uPL might also be a promising candidate for training networks for MR motion correction what will be investigated in future work.’*

What is the practical application implication of these results? You mention reduced imaging time, but to what extent would this allow that? You also mention the ability to use less computationally expensive denoising, but there is not an investigation of comparative performance in that regard, so it feels out-of-context. What specific advantage is gained from using this approach?

*The proposed approach can be used for eliminating noise from MR images containing line-like structures. This would e.g. allow shorter scan times. However, to what extent this can be used in the clinic or for plant research, still needs to be investigated. For the use in a clinical setting, the network needs to be applied on a variety of images including data from healthy and sick subjects. Likely, also additional methods indicating e.g. the network uncertainty (i.e. ensemble models) need to be employed before a clinical implementation can be realized. For the use in plant science, it needs to be very carefully investigated for which kind of plants the method can be used. However, these investigations will be performed in future work. The proposed approach demonstrates that the uPL is a promising candidate for MR image denoising of line-like structures and should definitely be considered in a clinical application. We mention this limitation for root data already in the discussion: ‚With a best mean SSIM of 0.87 the denoising results for our root dataset still need improvement to allow for reduced measurement time in the targeted application.‘ And added a part regarding the MRA dataset: ‚However, to apply the proposed approach in a clinical setting, our denoising framework needs to be verified on MRA data including healthy and unhealthy subjects (i.e. patients with e.g. an aneurysm). In a clinical scenario, it needs to be verified that denoising does not suppress important clinical information. This will be done in future studies based on this work.‘*

Comments:

Lines 70-72 “For both datasets, four levels of Rician noise were artificially added to assess the impact of the loss functions for different signal-to-noise ratios (1%, 5%, 10%, and 20% noise added)”

I would be interested to see images with the various noise levels. It may be helpful for contextualizing the quality of denoising. I do not see these in your figures containing the GT images and denoised images.

*Thank you for this remark. We added a new Figure (Figure 2) displaying an example root and MRA image with different noise levels.*

Line 181 “The training parameters were chosen as they lead to the overall best performance in the validation sets”

Could you expand a bit on what you mean by validation sets? What exactly did you do to determine that these were the best parameters? I don’t think you need a lot of extra detail here, but it would be nice to understand how you came to these training parameters, since these are fairly important for the overall performance of the network and since you are investigating other parameters in depth.

*We thank the reviewer for this comment. As described in section ‘Datasets’, we split datasets in 3 parts, a training, a validation, and a test set. As usual in machine learning experimentation, the validation set was used for validation purposes during training, allowing e.g. for parameter tuning. Tests were run on the test set. We added the following paragraph to the manuscript: ‚For this purpose, networks were trained for 10.000, 15.000, 20.000, ... 50.000 iterations, batch size was set to 4, 8, 16, 24, and 32, and the learning rate was set to 0.001,0.002, ... 0.005. All possible combinations of these parameters were investigated and the parameters leading to the best performance in the above defined validation datasets were selected.’*

Lines 190-192 "A cube of size 52 × 52 × 52 was cropped from the root data. 190 As the roots grow from top to bottom, the cube is cropped from the upper middle part of the image. A cube of size 68 × 68 × 68 is cropped from the middle of the MRA"

Is there a reason for these cube sizes? What does middle and upper middle mean in this context? Where all sample cubes taken from the same coordinates?

*We thank the reviewer for this comment. The objective of calculating the evaluation metrics on a part from the image was to calculate the metrics on regions only containing important information and no background. Therefore, we chose the cube size such that as little background as possible was present in the image part. We also tested with slightly varying cube sizes and did not observe notable changes. For the MRA images, the cropped part does not contain any background and by placing the cube on the location of the center of the root, as little background as possible is included. As the MRA images contain different numbers of z-slices, the cube was in these cases not placed at the same coordinates. For the MR-root images, the same coordinates were always chosen. We changed the text to make this more clear: ‚A cube of size 52×52×52 was cropped from the root data. As the roots grow from top to bottom and because the plant is always placed in the center of the scanner, the image was cropped such that the upper, middle image part was always present in the evaluation. I.e. the image was cropped from z-slices 0 - 52, and x-, and y-slices from 70-132. A cube of size 68×68×68 is cropped from the middle of the MRA images as these images contain important information mainly in the image center i.e. the center of the image was determined and a cube with the mentioned size was cropped such that the center of the cube aligned with the center of the image.‘*

Figures 2, 3, 4

I think these figures could use better captions – they are missing some key info. For example, in Fig 2, you do not mention the noise level, or what exactly is being displayed in the caption (but then describe it in text). This should be moved to the caption for easier reference. The same holds true for figures 2 & 3. I see that this info is included elsewhere, but as a reader, it is difficult to keep track of what is being displayed.

*We thank the reviewer for this comment. We changed all captions accordingly.*

Figure 4

There are arrows (presumably pointing to features of interest) but they are not explained. Why have you chosen to highlight these points?

*Thank you for this remark. We highlighted these points to mark differences across images. We added this to the Figure captions.*

Tables

Why are some values underlined? It would be helpful to provide a legend/explanation in the caption for this. In general, I think the table and figure captions could be revised for clarity and content.

*We thank the reviewer for pointing this out. We changed the table captions to improve clarity.*

Line 253 “Denoising network architecture vs. loss function for different noise levels”

Why only use L1 here as a comparison?

*As we investigated a large number of parameters and network architectures, we decided to only include the results of the L1-loss to keep a better overview. The general trend that the uPL loss outperforms other loss networks is already shown in the previous experiments and would not add valuable information here.*

Lines 328-334

This section of text feels somewhat out of place.

*We changed the text to: ‚None of the tested denoising network/loss function combinations allow to recover very fine roots, even though the performance increase using our uPL is considerable. In consequence, as of now, plants containing very fine roots need to be scanned for a longer time than plants with thick roots. Therefore, before implementing the trained networks on a plant root scanner, it needs to be verified for which plants (i.e. which root thickness) reduced image time in combination with a denoising network can be used without losing fine details.’*

Lines 335-336 “However, our results demonstrate the benefit of the untrained Perceptual Loss for both 3D datasets and all noise levels.”

This final sentence should be reworked a bit. It doesn’t flow particularly well with the prior paragraph, and I think that a final statement/claim like this should be given more attention and justification.

*Thank you for this comment. We changed the text to: ‚All in all, our results demonstrate that the untrained Perceptual Loss can be used in image denoising networks. To what extent it can be used for image enhancement in a clinical scenario, needs to be determined in future work. However, the untrained perceptual loss should definitely considered when training neural networks on MR images displaying fine, line-like structures.’*

Reviewer 4:

The topic is relevant and may be of interest to a broad range of the journal's readers. However, this reviewer has some major concerns about the paper.

1) The abstract does not highlight the specifics of the research or findings but contains too much background information. It is good to provide some specifics (e.g., sample size, dataset size, numbers from results, etc.).

*We thank the reviewer for pointing this out. We changed the abstract accordingly. We added the following parts: ‚In this work, we concentrate on image denoising of MR images containing line-like structures such as roots or vessels. In particular, we investigate if the special characteristics of these datasets (connectivity, sparsity) benefit from the use of special loss functions for network training.’; ‚In this study, 536 MR images of plant roots in soil and 450 MRA images are included. The plant root dataset is split to 380, 80, and 76 images for training, validation, and testing. The MRA dataset is split to 300, 50, and 100 images for training, validation, and testing.’; ‚Our results are compared with the frequently used L1 loss for different network architectures. We observe, that our uPL outperforms conventional loss functions such as the L1 loss or a loss based on the Structural Similarity Index Metric (SSIM). For MRA images the uPL leads to SSIM values of 0.93 while L1 and SSIM loss led to SSIM values of 0.81 and 0.88, respectively. The uPL network*’*s initialization is not important (e.g. for MR root images SSIM differences of 0.01 occur across initializations, while network depth and pooling operations impact denoising performance slightly more (SSIM of 0.83 for 5 convolutional layers and kernel size 3 vs. 0.86 for 5 convolutional layers and kernel size 5 for the root dataset).‘*

2) The discussion of the results does not highlight the strengths and weaknesses of the proposed approach.

*Thank you for pointing this out. We changed the discussion accordingly and added the following part: ‚None of the tested denoising network/loss function combinations allow to recover very fine roots, even though the performance increase using our uPL is considerable. In consequence, as of now, plants containing very fine roots need to be scanned for a longer time than plants with thick roots. Therefore, before implementing the trained networks on a plant root scanner, it needs to be verified for which plants (i.e. which root thickness) reduced image time in combination with a denoising network can be used without losing fine details. Results are considerably better for the MRA dataset. With SSIM values between 0.99 and 0.93 for the lower noise levels, the proposed networks might be used for image denoising in a clinical setting. However, to apply the proposed approach in a clinical setting, our denoising framework needs to be verified on MRA data including healthy and unhealthy subjects (i.e. patients with e.g. an aneurysm). In a clinical scenario, it needs to be verified that denoising does not suppress important clinical information. This will be done in future studies based on this work.*

*Our study has several limitations. First, all networks were trained and applied on data acquired at the same scanner. In future work, we will train and test our approach on data from different scanners yielding different image characteristics. Second, the MRA dataset used in this study yields exclusively images from healthy subjects. In future work, we will apply the networks also to subjects with vessel abnormalities. MR images of human subjects also suffer from motion artifacts. The proposed uPL might also be a promising candidate for training networks for MR motion correction what will be investigated in future work. All in all, our results demonstrate that the untrained Perceptual Loss can be used in image denoising networks. To what extent it can be used for image enhancement in a clinical scenario, needs to be determined in future work. However, the untrained perceptual loss should definitely considered when training neural networks on MR images displaying fine, line-like structures.’*

3) I suggest adding a clear research objective and research questions in the introduction section and specify what the main research problem or hypothesis is addressed.

*We thank the reviewer for this important point. We added the following part to the introduction: ‚ As displayed in Table 1, most recent studies addressing image denoising of MR brain or MRA images focus on the most adequate network architecture while using conventional loss functions such as L1 and/or SSIM-loss. Therefore, the aim of this work is to investigate if the uPL can be used beneficially for 3D images containing line-like structures. The research objective is to assess the impact of uPL network parameters on the results. We are especially interested if also small, simple networks can be used in the uPL.’*

4) Please add more recent references. Certainly, there has been more recent (within the last two years) research on this topic published in information science and/or computer science outlets. An academic search on the topic (using keywords from the manuscript's title) shows that there is recent work in this area. Therefore, authors must update their literature review.

*Thank you for this comment. We added a Table including recent work (Table 1).*

5) Starting from the previous works, I suggest introducing a table to summarize the most recent works and to highlight the novelty of the proposed work.

*Thank you for pointing this out. We added a part to the introduction (see answer to question 3).*

6) The manuscript only provides the structure of network, it is necessary to explain why these networks can work effectively and to show the concrete parameters.

*Unfortunately, it is not 100% clear to us to which network structure the reviewer is pointing. We assume that he/she refers to the Transformer network we are using (and not the loss networks which we extensively investigate and explain). The motivation to use the Transformer network is, that it was the best-performing architecture available for (2d) image denoising, when we performed the investigation. In addition, we added the missing number of feature maps to the manuscript. The Transformer part reads now: ‚In this network, multi-head* ’*transposed*’ *attention (MDTA) blocks are introduced applying attention across feature dimensions rather than across spatial dimensions. The used Transformer networks starts with a convolutional layer with kernel size 3 and 8 output feature maps. The convolutional layer is followed by a MDTA block. Before feeding the data to the MDTA blocks, the images are resized to the size (image height x image width x image depth) x number of channels. After the MDTA blocks follow feed-forward blocks consisting of two convolutional layers (with kernel size 3 and 16, and 32 output feature maps) and a gating layer.'*

7) Although a novel approach might be allowed, it is required to provide technical details of the proposed methods as much as possible and in-depth explanations of method selection process.

*We added the parameters of the Transformer network and hope this covers the missing details noted. From all other networks, parameters are provided. Also the loss functions are described in detail. If we should still be missing something, please let us know.*

8) While acquiring the image sometimes the quality may be poor due to blurring which may be caused due to motion artefact. How this problem may be tackled in the proposed scheme?

*The purpose of this work is the application of uPL for image denoising. We do not mention or tackle motion correction as this is a different problem of MR images. Additionally, the root data does not suffer from any motion. Therefore, the proposed method does not cover motion correction. However, for the implementation of motion correction networks, the uPL might also be a good loss function – we of course do not claim this as a finding in the paper as this needed to be investigated in future work. We added the following statement to the discussion:*

*‚MR images of human subjects also suffer from motion artefacts. The proposed uPL might also be a promising candidate for training networks for MR motion correction. However, this needs to be investigated in future work.‘*

9) The authors should need to site all equations and Figures in text.

*Thank you for pointing this out. We added the missing reference of Figure 4 to the text.*

10) It is suggested to conduct statistical tests. For example, see Section 4.5 in paper [a].

[a] Biswas, S., Mostafiz, R., Uddin, M.S. and Paul, B.K., 2024. XAI-FusionNet: Diabetic foot ulcer detection based on multi-scale feature fusion with explainable artificial intelligence. *Heliyon*, *10*(10), p.e31228.

*We ran each training setting various times and calculate variances across the runs. This demonstrates that the uPL performs consistently better than the other loss functions, where mean values are considerably different (i.e. 0.86 vs. 0.9) and variances are small enough to get an intuition on significance and effect size without proper statistical testing. That said, we believe that while being generally a good idea, formal statistical testing does not add extra information in this case.*

11) I think, the authors should introduce a comparison table to compare the proposed work with other existing works.

*We thank the reviewer for this comment. We compare the proposed loss function with existing loss functions and compare the results (see Table 2 ). Other existing works do not use the same datasets what makes a comparison difficult. However, as demonstrated our loss function shows clear advantages when compared with ‚state-of-the-art‘ loss functions.*

12) The reference list needs tidying up, as there are references missing items or formatting issues. Please be consistent with the formatting and use some standard formatting style.

*Done*
